# Prognostic Impact of Baseline Neutrophil-to-Lymphocyte Ratio and Its On-Treatment Change on Survival Outcomes in Advanced Small-Cell Lung Cancer: A Retrospective Analysis

**DOI:** 10.3390/cancers18040671

**Published:** 2026-02-18

**Authors:** Masashi Ishihara, Hao Chen, Reina Asaga, Hikaru Suzuki, Shinichiro Yamamoto, Maju Kawamoto, Hitoshi Hoshiya, Hiroki Kazahari, Ryosuke Ochiai, Shigeru Tanzawa, Takeshi Honda, Yasuko Ichikawa, Kiyotaka Watanabe, Nobuhiko Seki

**Affiliations:** 1 Division of Medical Oncology, Department of Internal Medicine, Teikyo University School of Medicine, Itabashi 173-8606, Japan; m.ishihara@med.teikyo-u.ac.jp (M.I.); asaga.reina.as@teikyo-u.ac.jp (R.A.); suzuki.hikaru.xj@teikyo-u.ac.jp (H.S.); yamamoto.shinichirou.fa@teikyo-u.ac.jp (S.Y.); kawamoto.maju.is@teikyo-u.ac.jp (M.K.); hoshiya.hitoshi.of@teikyo-u.ac.jp (H.H.); ryo7132003@med.teikyo-u.ac.jp (R.O.); s.tanzawa@med.teikyo-u.ac.jp (S.T.); thonda@med.teikyo-u.ac.jp (T.H.); icchi@med.teikyo-u.ac.jp (Y.I.); kiyowata@med.teikyo-u.ac.jp (K.W.); 2 Chemotherapy Center, Yokohama Minami Kyosai Hospital, Yokohama 236-0037, Japan; chinsmd@gmail.com; 3 Division of Respiratory Medicine, The Fraternity Memorial Hospital, Sumida 130-8587, Japan; kazahari-hiroki@douai.jp

**Keywords:** neutrophil-to-lymphocyte ratio, small cell lung cancer, biomarkers, systemic inflammation

## Abstract

Background: Reliable and readily accessible prognostic biomarkers for extensive-stage small-cell lung cancer (ES-SCLC) are still lacking. The neutrophil-to-lymphocyte ratio (NLR), a marker of systemic inflammation, has shown prognostic relevance in several malignancies; however, its dynamic changes during treatment have not been well characterized in SCLC. Methods: We retrospectively analyzed patients with ES-SCLC who received systemic chemotherapy between January 2010 and December 2024. Baseline NLR was calculated within 7 days before first-line treatment, and on-treatment NLR was assessed at 6 weeks. A predefined NLR cutoff value of 5 was applied, and changes in NLR (ΔNLR) were defined as the difference between 6-week and baseline values. Associations with time to treatment failure (TTF) and overall survival (OS) were evaluated. Results: A total of 176 patients were enrolled. High baseline NLR (≥5) was significantly associated with shorter TTF and OS (both *p* < 0.01). An increase in NLR during treatment (ΔNLR ≥ 0) was significantly associated with poorer OS. Combined assessment of baseline NLR and ΔNLR identified distinct prognostic groups, with patients exhibiting both high baseline NLR and ΔNLR ≥ 0 demonstrating markedly poor survival. In multivariate analyses, baseline NLR, ΔNLR, performance status, and immune checkpoint inhibitor combination therapy were independent predictors of survival. Baseline NLR analyzed as a continuous variable showed a significant inverse correlation with TTF and OS. Conclusions: Combined evaluation of baseline NLR and its on-treatment change provides improved prognostic stratification in patients with ES-SCLC.

## Simple Summary

Extensive-stage small-cell lung cancer is an aggressive disease, and predicting patient outcome remains difficult in clinical practice. Simple and widely accessible biomarkers are needed to enable clinicians to better stratify prognosis during the course of treatment. One such marker is the neutrophil-to-lymphocyte ratio (NLR), which can be calculated from routine blood tests and reflects inflammation in the body. In this study, we assessed whether baseline and 6-week NLR were associated with survival in patients with ES-SCLC treated with chemotherapy. We found that patients with higher baseline values and those with increases within six weeks of treatment initiation had poorer survival outcomes. These results suggest that early monitoring of this blood-based marker may help identify patients at higher risk and support treatment decision-making using routinely collected clinical data.

## 1. Introduction

Extensive-stage small-cell lung cancer (ES-SCLC) is a highly proliferative and metastatic disease [1]. In recent years, the advent of immune checkpoint inhibitors (ICIs) targeting programmed cell death receptor 1 (PD-1) or programmed death-ligand 1 (PD-L1) has led to improvements in survival, as demonstrated in the IMpower133 and CASPIAN trials [2,3]. While the PD-L1 expression is a useful marker with response of immune check point inhibitors (ICIs), its expression is low in SCLC as tumor characteristic [4,5,6]. In ES-SCLC, tumor mutation burden (TMB) is similarly not practical, and therefore no useful biomarker has been established [7]. More recently, transcription factor–based molecular subtypes defined by ASCL1, NEUROD1, POU2F3, and YAP1 have provided important biological insights into SCLC heterogeneity [8]. However, these approaches rely on tumor tissue and specialized molecular analyses that are not always feasible in routine practice. Therefore, simple and broadly accessible biomarkers remain of clinical interest.

Cancer-related systemic inflammation plays a crucial role in tumor progression and survival across malignancies [9,10,11,12]. In SCLC, several studies have demonstrated that elevated baseline NLR is associated with inferior survival outcomes [13,14]. A recent meta-analysis encompassing more than 3000 patients confirmed that higher NLR was consistently associated with worse overall and progression-free survival, supporting its role as a systemic inflammation–based prognostic marker [15]. Inflammation-based prognostic models such as the MDACC scoring system, which incorporates NLR and related markers, have also been evaluated in clinical SCLC cohorts and shown to provide additional risk stratification [16].

However, variability in the selection of NLR cutoff values across studies makes direct comparison challenging, as thresholds are often derived from cohort-specific receiver operating characteristic (ROC) analyses or median-based dichotomization. ROC curve–based approaches are statistically suboptimal for time-to-event endpoints because they do not adequately account for censoring and may yield unstable thresholds across cohorts [17,18,19]. In addition, although baseline NLR has been extensively evaluated, fewer studies have examined its early on-treatment changes, and the relative contribution of baseline and dynamic measures remains to be further explored. While composite inflammation-based models such as the MDACC score may provide broader biological assessment, simpler markers derived from routine laboratory data remain attractive for practical clinical application.

Therefore, in the present study, we focused on baseline NLR and its dynamic change during treatment as accessible biomarkers and evaluated their association with treatment durability and survival outcomes in patients with ES-SCLC receiving systemic chemotherapy in real-world practice.

## 2. Materials and Methods

### 2.1. Patients and Data Collection

Study subjects were selected from patients at Teikyo University Hospital in Japan. The patients with ES-SCLC who were treated with chemotherapy between January 2008 and December 2024 were included in this study. The progression of lung cancer was determined according to the 8th edition [17], and the following clinical information was collected: age, gender, smoking history, Eastern Cooperative Oncology Group performance status (ECOG-PS), use of immune-checkpoint inhibitors (Atezolizumab or Durvalumab), time to treatment failure (TTF), overall survival (OS). Complete blood counts with differential were obtained at baseline (within 7 days prior to treatment initiation) and at 6 weeks after treatment initiation. Absolute neutrophil and lymphocyte counts were obtained from routine blood tests, and the neutrophil-to-lymphocyte ratio (NLR) was calculated as the ratio of neutrophils to lymphocytes. Baseline NLR was defined as the pretreatment value, while the change in NLR (ΔNLR) was calculated as the difference between the 6-week post-treatment NLR and the baseline NLR.

### 2.2. Statistical Methods

Patients’ clinical characteristics were compared using Fisher’s exact test for categorical variables and the Wilcoxon rank-sum test for continuous variables. Survival curves for TTF and OS were estimated using the Kaplan–Meier method and compared using the log-rank test. Baseline NLR was analyzed as a categorical variable using a predefined cutoff value of 5. The cutoff value was selected based on prior clinical studies [12,18,19,20] rather than data-driven approaches such as ROC curve analysis. ROC-based determination was not applied because survival outcomes are subject to censoring, and optimal cutoff values derived from ROC analyses may vary across studies and patient populations.

Patients were stratified according to the change in NLR during treatment (ΔNLR < 0 vs. ≥0), and Kaplan–Meier analyses were performed to evaluate differences in TTF and OS between groups. For exploratory analyses, patients stratified by ΔNLR were further categorized according to NLR measured at 6 weeks after treatment initiation (NLR2), using a predefined cutoff value of 5.

Multivariate analyses for TTF and OS were conducted using Cox proportional hazards regression models. Variables considered clinically relevant were simultaneously entered into the multivariate models, and hazard ratios (HRs) with 95% confidence intervals (CIs) were calculated. Finally, correlations between baseline NLR values and survival outcome (TTF and OS) were assessed using Spearman’s rank correlation coefficient. All statistical tests were two-sided, and statistical significance was defined as a *p* value < 0.05. All statistical analyses were performed using EZR version1.70 (Saitama Medical Center, Jichi Medical University).

## 3. Results

### 3.1. Patient Characteristics

**Figure 1:**
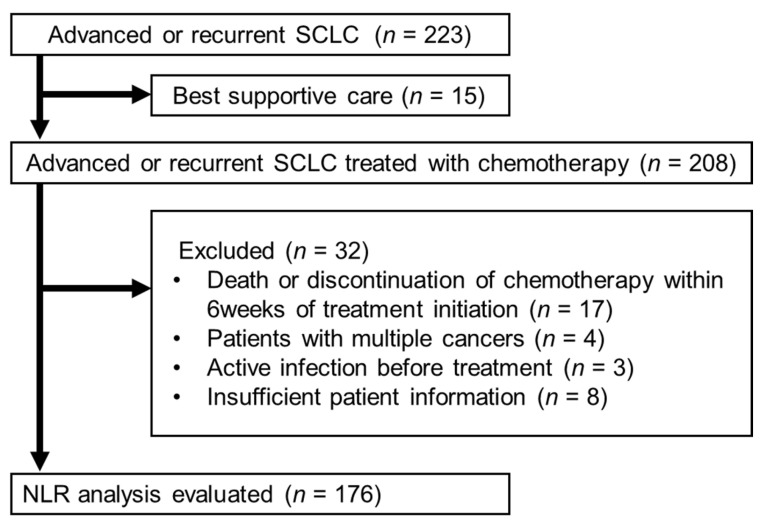
Flow diagram of patients through the study. SCLC, small-cell lung cancer; NLR, neutrophil to lymphocyte ratio.

As shown in Figure 1, 176 patients were enrolled. The characteristics of the study participants are summarized in Table 1. 176 patients were enrolled (131 men and 45 women; median age, 70.0 years [range, 37–86]). 160 patients (90.9%) were current or former smokers. Approximately two-thirds of the patients had a favorable ECOG performance status (PS 0–1), whereas 10.8% had a PS of 3 or higher. 54 patients (30.7%) received anti-PD-L1 antibody plus chemotherapy. CNS metastases were observed in 23.9% of patients. A total of 54 patients (30.7%) received immunotherapy (anti-PD-L1 inhibitors) in combination with systemic treatment. The median age differed between patients stratified by ΔNLR (*p* = 0.01), whereas no other baseline clinical characteristics differed between groups stratified by baseline NLR or ΔNLR.

### 3.2. Baseline NLR and Survival Outcomes

**Figure 2:**
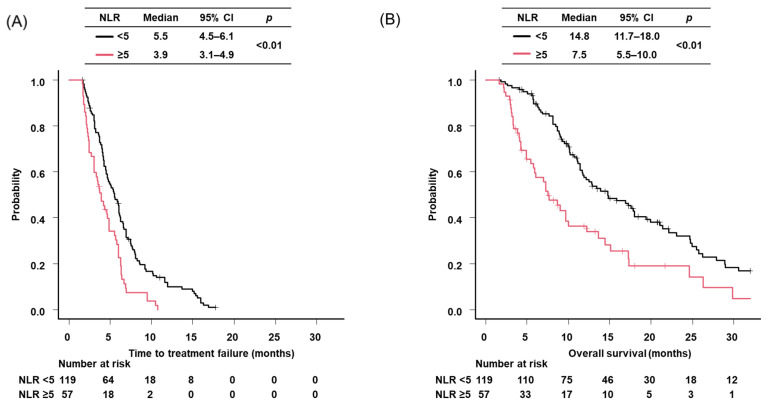
Kaplan-Maier survival curves of ES-SCLC patients by each index. (**A**) time to treatment failure of patients stratified by baseline NLR; (**B**) overall survival of patients stratified by baseline NLR. NLR, neutrophil to lymphocyte ratio; CI, confidence interval.

In overall, median TTF and OS were 4.9 months (95% CI: 4.2–5.6) and 12.2 months (95% CI: 10.2–14.8) (Supplementary Figure S1). Median TTF and OS were 5.5 months (95% CI: 4.5–6.1) and 14.8 months (95% CI: 11.7–18.0) for patients with NLR < 5, respectively, and 3.9 months (95%CI: 3.1–4.9) and 7.5 months (95% CI: 5.5–10.0) for patients with NLR ≥ 5, respectively (both *p* < 0.01) (Figure 2). The multivariate analysis demonstrated that poor PS (both ECOG PS 3 [HR: 1.65, 95% CI: 1.04–2.89] and 4 [HR: 2.82, 95% CI: 1.56–6.89]), ICI combination therapy (HR: 0.67, 95% CI: 0.45–0.93) and baseline NLR (≥5) (HR: 1.93, 95% CI: 1.37–2.72) were significant independent predictors of TTF (Table 2). With respect to OS, the same variables remained independent predictors, with HRs of 1.29 (95% CI: 1.07–2.36) for ECOG PS 3, 3.66 (95% CI: 1.11–12.0) for ECOG PS 4, 0.56 (95% CI: 0.35–0.84) for ICI combination therapy, and 2.21 (95% CI: 1.50–3.26) for baseline NLR ≥ 5 (Table 2).

Exploratory analyses using alternative cutoffs showed consistent trends. At a cutoff of 4, patients with NLR ≥ 4 had shorter TTF and OS than those with NLR < 4 (TTF: 4.3 vs. 5.6 months; OS: 9.0 vs. 17.6 months; n = 77 vs. 99; both *p* < 0.01). Similarly, at a cutoff of 3, patients with NLR ≥ 3 also showed shorter TTF and OS compared with those with NLR < 3 (TTF: 4.5 vs. 6.0 months; OS: 10.2 vs. 18.0 months; n = 107 vs. 69; both *p* < 0.01).

### 3.3. ΔNLR and Survival Outcomes

**Figure 3:**
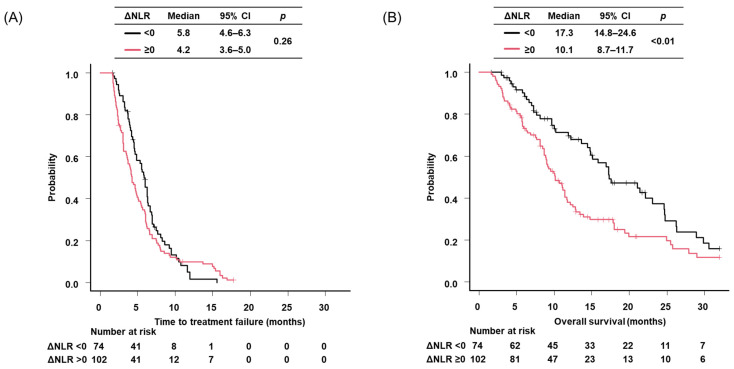
Kaplan-Maier survival curves of ES-SCLC patients by each index. (**A**) time to treatment failure of patients stratified by ΔNLR; (**B**) overall survival of patients stratified by ΔNLR. NLR, neutrophil to lymphocyte ratio; CI, confidence interval.

Median TTF and OS were 5.8 months (95% CI: 4.6–6.3) and 17.3 months (95% CI: 14.8–24.6), for patients with ΔNLR < 0, respectively, and 4.2 months (95%CI: 3.6–5.0) and 10.1 months (95% CI: 8.7–11.7), for patients with ΔNLR ≥ 0, respectively with *p* = 0.26 for TTF, and *p* < 0.01 for OS, respectively (Figure 3). The multivariate analysis demonstrated that poor PS (both ECOG PS 3 [HR: 1.74, 95% CI: 1.04–2.89] and 4 [HR: 3.44, 95% CI: 1.75–7.99]) and ICI combination therapy (HR: 0.67, 95% CI: 0.49–0.98) were independent predictors of TTF, whereas ΔNLR was not identified as an independent predictor of TTF (Table 3). With respect to OS, poor PS, ICI combination therapy, and ΔNLR (≥0) remained independent predictors, with HRs of 1.84 (95% CI: 1.19–2.86) for ECOG PS 3, 6.01 (95% CI: 1.79–20.2) for ECOG PS 4, 0.57 (95% CI: 0.35–0.93) for ICI combination therapy, and 1.93 (95% CI: 1.33–2.84) for ΔNLR ≥ 0 (Table 3).

To further assess the association between ΔNLR and clinical outcomes, patients were stratified according to NLR measured at 6 weeks after treatment initiation (NLR2), using a cutoff value of 5. Among patients with ΔNLR ≥ 0, those with NLR2 < 5 showed significantly longer TTF and OS than those with NLR2 ≥ 5 (Supplementary Figure S2A,B). In contrast, among patients with ΔNLR < 0, no significant difference in TTF was observed regardless of NLR2 status, whereas OS was significantly longer in patients with NLR2 < 5 than in those with NLR2 ≥ 5 (Supplementary Figure S2C,D).

### 3.4. Baseline NLR and ΔNLR with Survival Outcomes

**Figure 4:**
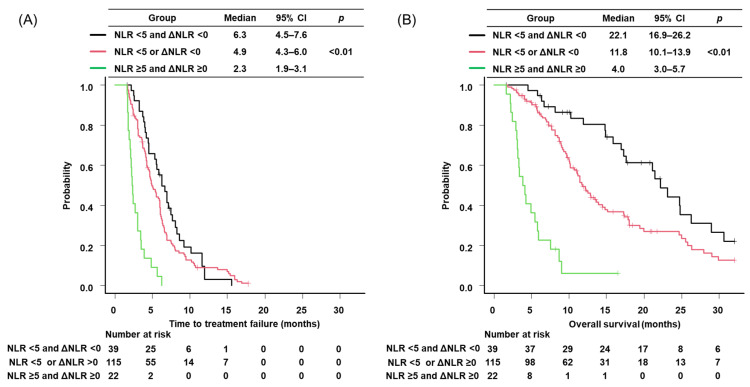
Kaplan-Maier survival curves of ES-SCLC patients by each index. (**A**) time to treatment failure of patients stratified by baseline NLR and ΔNLR; (**B**) overall survival of patients stratified by NLR and ΔNLR. NLR, neutrophil to lymphocyte ratio; CI, confidence interval.

To assess the combined prognostic impact of baseline NLR and ΔNLR, patients were classified according to two factors: baseline NLR ≥ 5 and ΔNLR ≥ 0. Patients with NLR < 5/ΔNLR ≥ 0 (n = 80) and those with NLR ≥ 5/ΔNLR < 0 (n = 35) showed comparable TTF and OS (Supplementary Figure S3). Accordingly, these two categories were combined into a single intermediate-risk group for the main three-group analysis. Based on the number of risk factors, patients were divided into three groups: group NLR < 5 and ΔNLR < 0 (no factors), group NLR < 5 or ΔNLR < 0 (one factor), and group NLR ≥ 5 and ΔNLR ≥ 0 (two factors). Group NLR < 5 and ΔNLR < 0 included 39 patients. Group NLR < 5 or ΔNLR < 0 comprised 115 patients. Group NLR ≥ 5 and ΔNLR ≥ 0 included 22 patients. Median TTF was 6.3 months (95% CI, 4.5–7.6) in group NLR < 5 and ΔNLR < 0, 4.9 months (95% CI, 4.3–6.0) in group NLR < 5 or ΔNLR < 0, and 2.3 months (95% CI, 1.9–3.1) in group NLR ≥ 5 and ΔNLR ≥ 0 (Figure 4A). Median OS was 22.1 months (95% CI, 16.9–26.2), 11.8 months (95% CI, 10.1–13.9), and 4.0 months (95% CI, 3.0–5.7), respectively (both *p* < 0.01) (Figure 4B). The multivariate analysis demonstrated that poor PS (both ECOG PS 3 [HR: 1.90, 95% CI: 1.09–3.31] and 4 [HR: 2.10, 95% CI: 1.65–7.81]), ICI combination therapy (HR: 0.66, 95% CI: 0.46–0.94) and high baseline NLR and ΔNLR (HR: 6.13, 95% CI: 3.45–10.9) were independent predictors of TTF (Table 4). With respect to OS, poor PS and ICI combination therapy remained independent factor, with HR of 1.76 (95% CI: 1.13–2.76) for ECOG PS 2, 1.91 (95% CI: 1.23–3.16) for ECOG PS 3, 6.73 (95% CI: 1.99–22.7) for ECOG PS 4. Moreover, low baseline NLR and high ΔNLR (≥0) or high baseline NLR and low ΔNLR (<0), and both high baseline NLR and high ΔNLR (≥0) remained independent predictors, with HRs of 2.14 (95% CI: 1.34–3.42) for group NLR < 5 or ΔNLR < 0, 12.9 (95% CI: 6.59–25.0) for group NLR ≥ 5 and ΔNLR ≥ 0 (Table 4).

### 3.5. Association of Continuous NLR Values with Overall Survival

**Figure 5:**
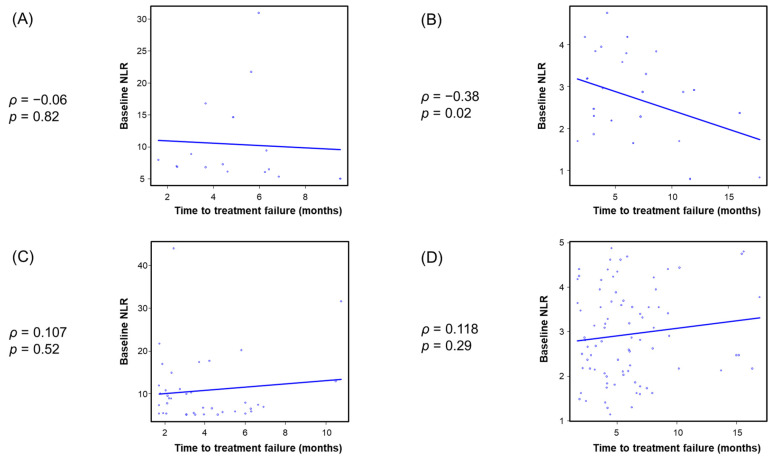
Association between baseline NLR and TTF in patients treated with immune checkpoint inhibitor-based combination therapy with (**A**) high NLR and (**B**) low NLR, and in those treated with cytotoxic chemotherapy alone with (**C**) high NLR and (**D**) low NLR. NLR, neutrophil to lymphocyte ratio.

**Figure 6:**
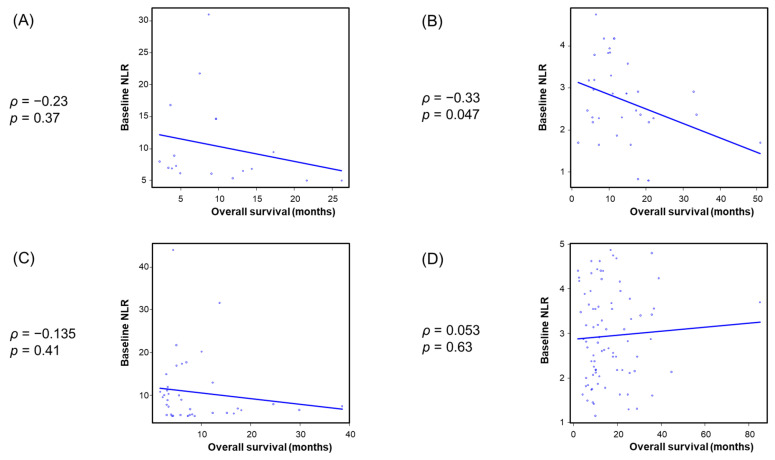
Association between baseline NLR and OS in patients treated with immune checkpoint inhibitor–based combination therapy with (**A**) high NLR and (**B**) low NLR, and in those treated with cytotoxic chemotherapy alone with (**C**) high NLR and (**D**) low NLR. NLR, neutrophil to lymphocyte ratio.

Spearman’s rank correlation analysis showed an inverse correlation between baseline NLR and both TTF and OS in the ICI-based combination and cytotoxic chemotherapy–alone groups (Supplementary Figure S4). To further examine this association, patients were stratified by both treatment and baseline NLR using a predefined cutoff value of 5, resulting in four subgroups: ICI-based combination therapy with low NLR (<5) and high NLR (≥5), cytotoxic chemotherapy alone with low NLR (<5) and high NLR (≥5). As shown in Figure 5 and Figure 6, among patients who treated with ICI-based chemotherapy, an inverse correlation between baseline NLR and both TTF and OS was observed in the low-NLR subgroup, whereas no significant correlation was detected in the high-NLR subgroup. Among patients treated with cytotoxic chemotherapy alone, no significant correlation between baseline NLR and both TTF and OS was observed after stratification by baseline NLR status.

## 4. Discussion

This study demonstrated that both baseline NLR and its early on-treatment dynamics were associated with survival outcomes in patients with ES-SCLC treated with systemic chemotherapy. Higher baseline NLR values were consistently associated with shorter TTF and OS, supporting the role of systemic inflammation as a prognostic determinant in SCLC. These findings are consistent with prior reports across lung cancer populations showing that elevated NLR reflects an unfavorable host inflammatory and immune milieu associated with poor prognosis [15,21,22,23].

Although NLR is inherently a continuous biomarker, threshold-based stratification remains clinically practical. A cutoff value of 5 has been widely adopted across lung cancer studies and has demonstrated consistent prognostic discrimination in both NSCLC and SCLC populations [21,22,23]. Meta-analytic evidence supports NLR = 5 as a robust survival threshold [22], and SCLC studies have shown survival separation across commonly used cutoffs, including 4 and 5 [23]. In our cohort, sensitivity analyses likewise showed preserved prognostic associations across alternative thresholds, with 5 providing the greatest separation. Thus, the use of a cutoff of 5 primarily reflects its ability to help identify a clinically meaningful poor-prognosis subgroup rather than a biologically discrete threshold.

In addition to baseline stratification, NLR and its early on-treatment dynamics may serve as a pragmatic framework for screening patients at increased risk of poor outcomes. Approximately 30% of patients had NLR ≥ 5, representing a clinically meaningful high-risk subgroup. Because NLR behaves as a continuous prognostic variable, threshold selection requires balancing risk enrichment against subgroup prevalence; lower cutoffs may dilute prognostic discrimination by including relatively favorable patients, whereas excessively high thresholds yield very small subgroups with limited clinical applicability. Within this context, NLR = 5 provides a practical compromise that enriches for poor-prognosis biology while retaining sufficient prevalence for clinical stratification. Importantly, incorporation of early NLR dynamics further refines risk identification beyond a single baseline measurement and may facilitate earlier recognition of patients with potentially limited treatment benefit or aggressive disease biology. In our analyses, baseline NLR showed more consistent associations with survival than ΔNLR alone, whereas dynamic change added complementary stratification when considered jointly, indicating that baseline NLR is the main prognostic factor, with on-treatment change providing additional stratification.

This dynamic risk-stratification framework is clinically relevant in ES-SCLC, given the substantial attrition following first-line therapy and the necessity for early treatment-sequencing decisions [24]. In the evolving post-platinum landscape, including the recent availability of tarlatamab in later-line settings, such early risk identification may support monitoring intensity and timely transition to subsequent therapies within an individualized treatment sequence [25]. Composite indices such as the MDACC score have been validated to provide comprehensive baseline risk stratification using multiple clinical and laboratory variables [16]. The present study addressed a different objective by focusing on NLR alone and assessing its early on-treatment change, thereby exploring its role as a practical and repeatedly assessable screening biomarker. Notably, the prognostic associations of NLR appeared more pronounced in patients receiving chemoimmunotherapy than in those treated with chemotherapy alone, which may reflect the closer linkage of NLR to host immune competence and tumor–immune interactions that modulate ICI efficacy [26,27,28].

The present study has several limitations. First, this was a retrospective analysis, and potential selection and confounding biases cannot be excluded. In particular, patients treated with cytotoxic chemotherapy alone may have had clinical factors precluding immunotherapy that independently influenced survival outcomes. Subgroup analyses were limited by reduced sample size, and the absence of significant associations in some strata should not be interpreted as lack of treatment benefit. Second, molecular subclassification of SCLC based on transcriptional or genomic features was not available because adequate tumor tissue and specialized immunohistochemical or molecular assays were not routinely obtained in this retrospective cohort. Therefore, potential relationships between SCLC molecular subtypes and systemic inflammatory status could not be assessed. Third, only a minority of patients received ICIs, limiting assessment of immune-related inflammatory effects, although the association between baseline NLR and survival was consistent across treatment groups. Finally, while prognostic extremes were identifiable (e.g., persistently elevated vs. low and decreasing NLR), most patients fell into an intermediate-risk category with less distinct survival separation, indicating the need for additional biomarkers to refine stratification in this heterogeneous population.

## 5. Conclusions

In conclusion, baseline NLR and its early on-treatment change provide complementary prognostic information in ES-SCLC. An NLR threshold of 5 identifies a clinically meaningful high-risk subgroup and, when combined with dynamic assessment, may serve as a simple framework for risk screening and treatment-strategy consideration in routine practice.

## Figures and Tables

**Table 1:** Characteristics of patients treated with chemotherapy.

Characteristics	Overall	Baseline NLR (*n*, %)			ΔNLR (*n*, %)		
		NLR < 5	NLR ≥ 5	*p*	ΔNLR < 0	ΔNLR ≥ 0	*p*
Patients	176	119	57	0.94	74	102	0.01
Median age [range], years	70.0 (37, 86)	70.0 (37, 86)	70.0 (49, 86)		67.5 (37, 86)	71.0 (49, 86)	
Age				0.95			0.18
<75 years	126	85 (71.4)	41 (71.9)		57 (77.0)	69 (67.6)	
≥75 years	50	34 (28.6)	16 (28.1)		17 (23.0)	33 (32.4)	
Sex				0.27			0.86
Female	45	27 (22.7)	18 (31.6)		18 (24.3)	27 (26.5)	
Male	131	92 (77.3)	39 (68.4)		56 (75.7)	75 (73.5)	
ECOG PS				0.06			0.73
0–1	118	87 (73.1)	31 (54.4)		47 (63.5)	71 (73.5)	
2	39	22 (18.5)	17 (29.8)		18 (24.3)	21 (20.6)	
3	16	9 (7.6)	7 (12.3)		7 (9.5)	9 (8.8)	
4	3	1 (0.8)	2 (3.5)		2 (2.7)	1 (1.0)	
Smoking status				0.31			0.50
Never	16	9 (7.6)	7 (12.3)		8 (10.8)	8 (78.4)	
Current/former	160	110 (92.4)	50 (87.7)		66 (89.2)	94 (92.2)	
CNS metastasis				0.71			0.15
No	134	92 (77.3)	42 (73.7)		52 (70.3)	82 (80.4)	
Yes	42	27 (22.7)	15 (26.3)		22 (29.7)	20 (19.6)	
ICI combination				0.86			0.17
No	122	83 (69.7)	39 (68.4)		47 (63.5)	75 (73.5)	
Yes	54	36 (30.3)	18 (31.6)		27 (36.5)	27 (26.5)	

NLR, neutrophil to lymphocyte ratio; ECOG PS, Eastern Cooperative Oncology Group Performance Status; CNS, central nervous system; ICI, immune checkpoint inhibitor.

**Table 2:** Multivariate analysis of associations of baseline NLR and survival.

Characteristics	Time to Treatment Failure		Overall Survival	
	HR (95% CI)	*p*	HR (95% CI)	*p*
Age (≥75 years)	0.91 (0.64–1.30)	0.60	1.41 (0.94–2.11)	0.09
Sex (male)	1.00 (0.70–1.44)	0.98	1.50 (0.98–2.31)	0.06
Smoke (current/former)	1.13 (0.91–1.53)	0.45	1.25 (0.91–1.69)	0.17
ECOG PS				
0–1	1 (ref)	–	1 (ref)	-
2	1.27 (0.85–1.88)	0.24	1.65 (0.77–2.54)	0.41
3	1.65 (1.04–2.89)	0.047	1.29 (1.07–2.36)	0.041
4	2.82 (1.56–6.89)	0.03	3.66 (1.11–12.0)	0.03
CNS metastasis (yes)	0.92 (0.63–1.35)	0.70	1.77 (0.70–1.61)	0.78
ICI combination (yes)	0.67 (0.45–0.93)	0.02	0.56 (0.35–0.84)	0.02
Baseline NLR (≥5)	1.93 (1.37–2.72)	<0.01	2.21 (1.50–3.26)	<0.01

HR, hazards ratio; CI, confidence interval; CNS, central nervous system; ECOG PS, Eastern Cooperative Oncology Group Performance Status; ref, reference category; ICI, immune checkpoint inhibitors; NLR, neutrophil to lymphocyte ratio.

**Table 3:** Multivariate analysis of associations of ΔNLR and survival.

Characteristics	Time to Treatment Failure		Overall Survival	
	HR (95% CI)	*p*	HR (95% CI)	*p*
Age (≥75 years)	0.89 (0.62–1.28)	0.54	1.50 (1.01–2.23)	0.044
Sex (male)	0.96 (0.67–1.37)	0.82	1.27 (0.83–1.95)	0.26
Smoke (current/former)	1.21 (0.96–1.59)	0.15	1.18 (0.78–1.79)	0.45
ECOG PS				
0–1	1 (ref)	–	1 (ref)	-
2	1.43 (0.91–2.13)	0.08	1.42 (0.78–2.59)	0.25
3	1.74 (1.04–2.89)	0.048	1.84 (1.19–2.86)	<0.01
4	3.44 (1.75–7.99)	0.043	6.01 (1.79–20.2)	<0.01
CNS metastasis (yes)	1.20 (0.82–1.76)	0.34	1.34 (0.89–2.01)	0.17
ICI combination (yes)	0.69 (0.49–0.98)	0.047	0.57 (0.35–0.93)	0.02
ΔNLR (≥5)	1.24 (0.90–1.71)	0.19	1.93 (1.33–2.84)	<0.01

HR, hazards ratio; CI, confidence interval; CNS, central nervous system; ECOG PS, Eastern Cooperative Oncology Group Performance Status; ref, reference category; ICI, immune checkpoint inhibitors; NLR, neutrophil to lymphocyte ratio.

**Table 4:** Multivariate analysis of associations of baseline NLR and ΔNLR and survival.

Characteristics	Time to Treatment Failure		Overall Survival	
	HR (95% CI)	*p*	HR (95% CI)	*p*
Age (≥75 years)	0.93 (0.65–1.34)	0.69	1.55 (1.04–2.31)	0.03
Sex (male)	0.95 (0.67–1.36)	0.79	1.31 (0.86–2.01)	0.21
Smoke (current/former)	1.32 (0.72–2.46)	0.38	1.54 (0.97–2.64)	0.07
ECOG PS				
0–1	1 (ref)	–	1 (ref)	–
2	1.35 (0.90–2.01)	0.14	1.76 (1.13–2.76)	0.01
3	1.90 (1.09–3.31)	0.02	1.91 (1.23–3.16)	0.01
4	2.10 (1.65–7.81)	0.03	6.73 (1.99–22.7)	<0.01
CNS metastasis (yes)	1.14 (0.77–1.67)	0.51	1.28 (0.85–1.93)	0.24
ICI combination (yes)	0.66 (0.46–0.94)	0.02	0.60 (0.37–0.97)	0.042
Group				
NLR < 5 and ΔNLR < 0	1 (ref)	–	1 (ref)	–
NLR < 5 or ΔNLR < 0	1.25 (0.84–1.54)	0.27	2.14 (1.34–3.42)	<0.01
NLR ≥ 5 and ΔNLR ≥ 0	6.13 (3.45–10.9)	<0.01	12.9 (6.59–25.0)	<0.01

HR, hazards ratio; CI, confidence interval; CNS, central nervous system; ECOG PS, Eastern Cooperative Oncology Group Performance Status; ref, reference category; ICI, immune checkpoint inhibitors.

## Data Availability

Data are contained within the article.

## Supplementary Materials

The following supporting information can be downloaded at: https://www.mdpi.com/article/10.3390/cancers18040671/s1, Figure S1: Kaplan–Meier curves for time to treatment failure (A) and overall survival (B) in the overall study population; Figure S2: Kaplan–Meier curves of time to treatment failure (TTF) and overall survival (OS) stratified by an NLR2 cutoff value of 5 according to on-treatment changes in NLR; ΔNLR ≥ 0, TTF (A) and OS (B); ΔNLR < 0, TTF (C) and OS (D); Figure S3: Kaplan–Meier curves for time to treatment failure (A) and overall survival (B) according to combined baseline neutrophil-to-lymphocyte ratio (NLR) and on-treatment NLR change (ΔNLR) Figure S4: Association between baseline NLR and time to treatment failure (TTF) or overall survival (OS) assessed by Spearman’s rank correlation analysis; TTF: ICI-based combination chemotherapy (A) and cytotoxic chemotherapy alone (B); OS: ICI-based combination chemotherapy (C) and cytotoxic chemotherapy alone (D).
