# Supplementary material for: Prognostic Impact of Baseline Neutrophil-to-Lymphocyte Ratio and Its On-Treatment Change on Survival Outcomes in Advanced Small-Cell Lung Cancer: A Retrospective Analysis
Source: Cancers (Basel). 2026 Feb 18;18(4):671. doi: 10.3390/cancers18040671 (PMC12939829; doi:10.3390/cancers18040671)
Supplement: Supplementary file 1 [file cancers-18-00671-s001.zip › cancers-4128585-supplementary.pdf]

Figure S1.

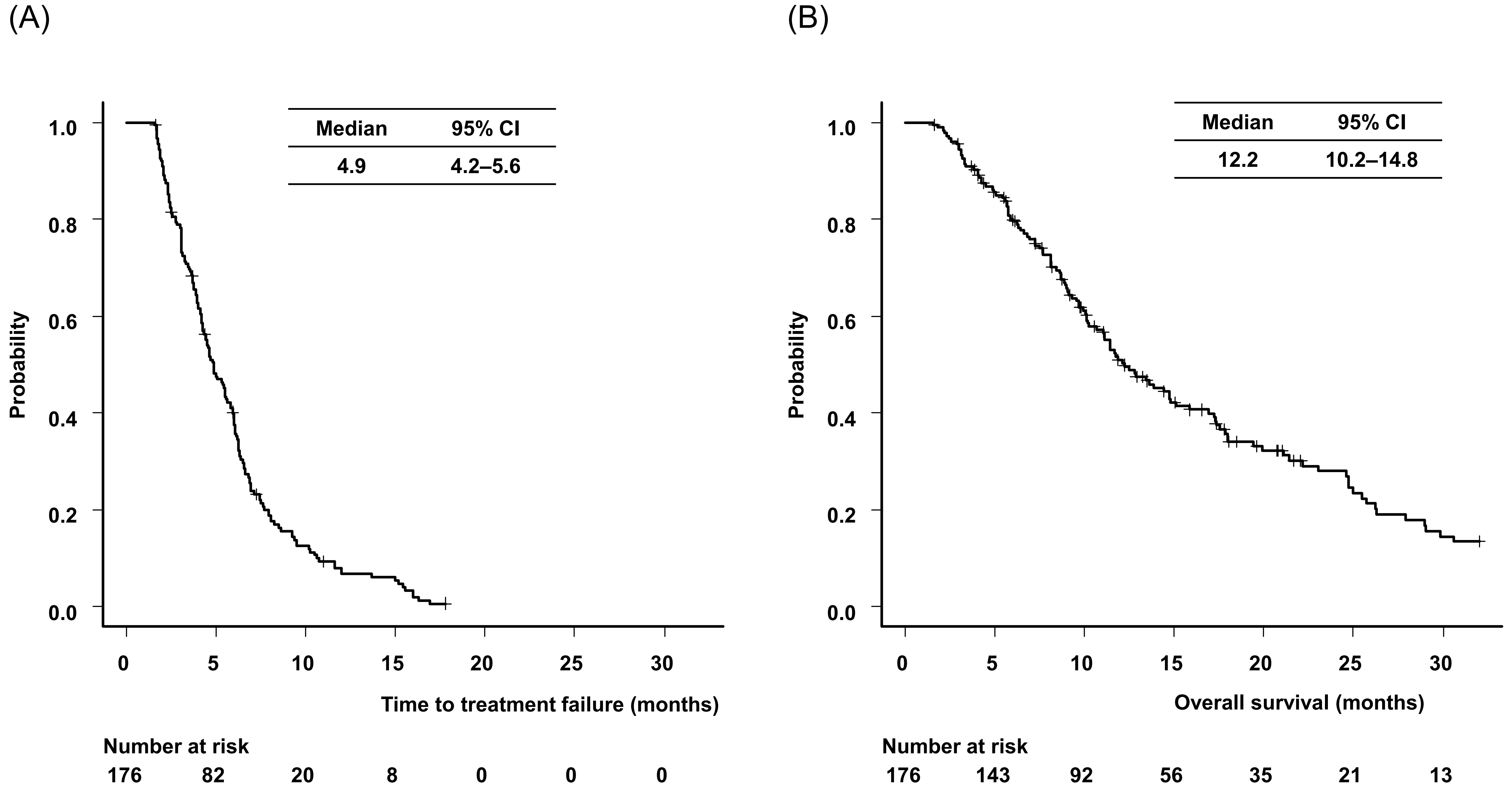

(A)
Median
95% CI
4.9
4.2–5.6
Probability
1.0
0.8
0.6
0.4
0.2
0.0
0
5
10
15
20
25
30
Time to treatment failure (months)
Number at risk
176
82
20
8
0
0
0
(B)
Median
95% CI
12.2
10.2–14.8
Probability
1.0
0.8
0.6
0.4
0.2
0.0
0
5
10
15
20
25
30
Overall survival (months)
Number at risk
176
143
92
56
35
21
13

Figure S2.

(A)

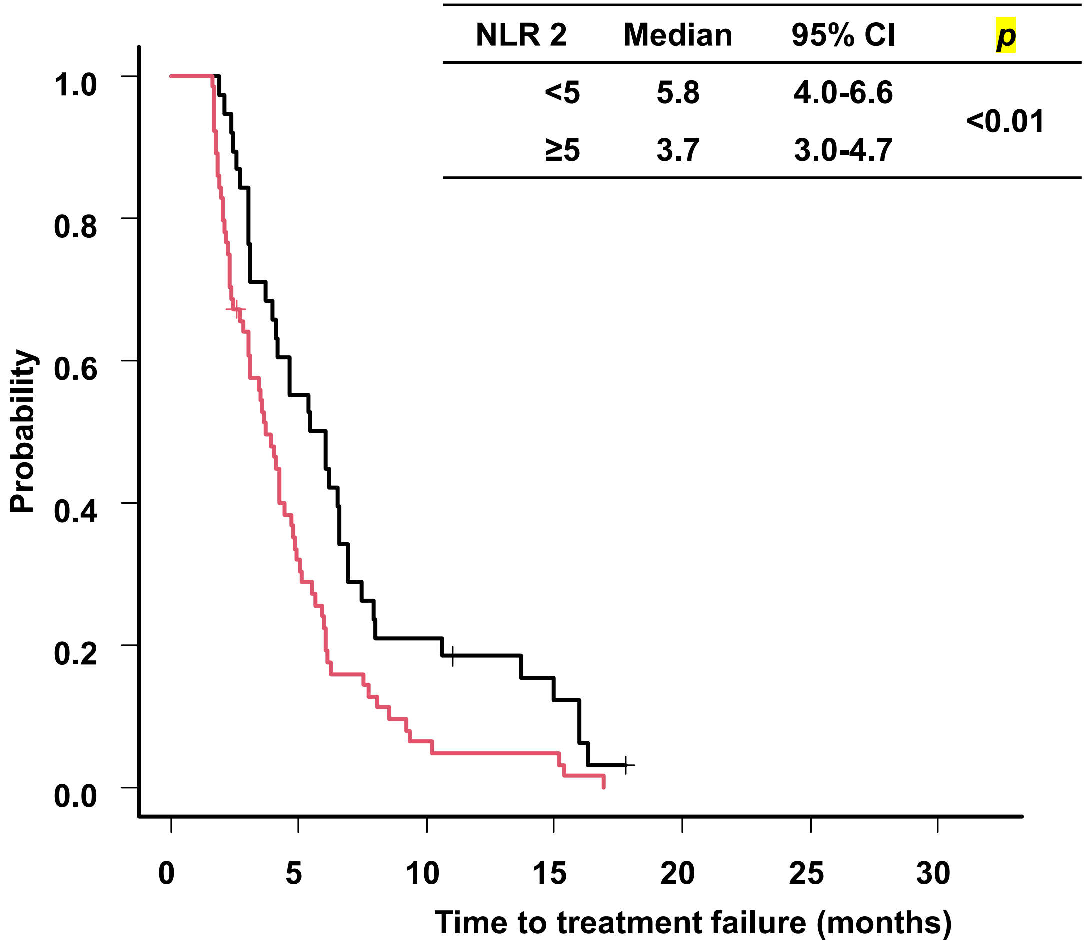

Number at risk

< 5	38	21	8	4	0	0	0
≥ 5	64	20	4	3	0	0	0

(B)

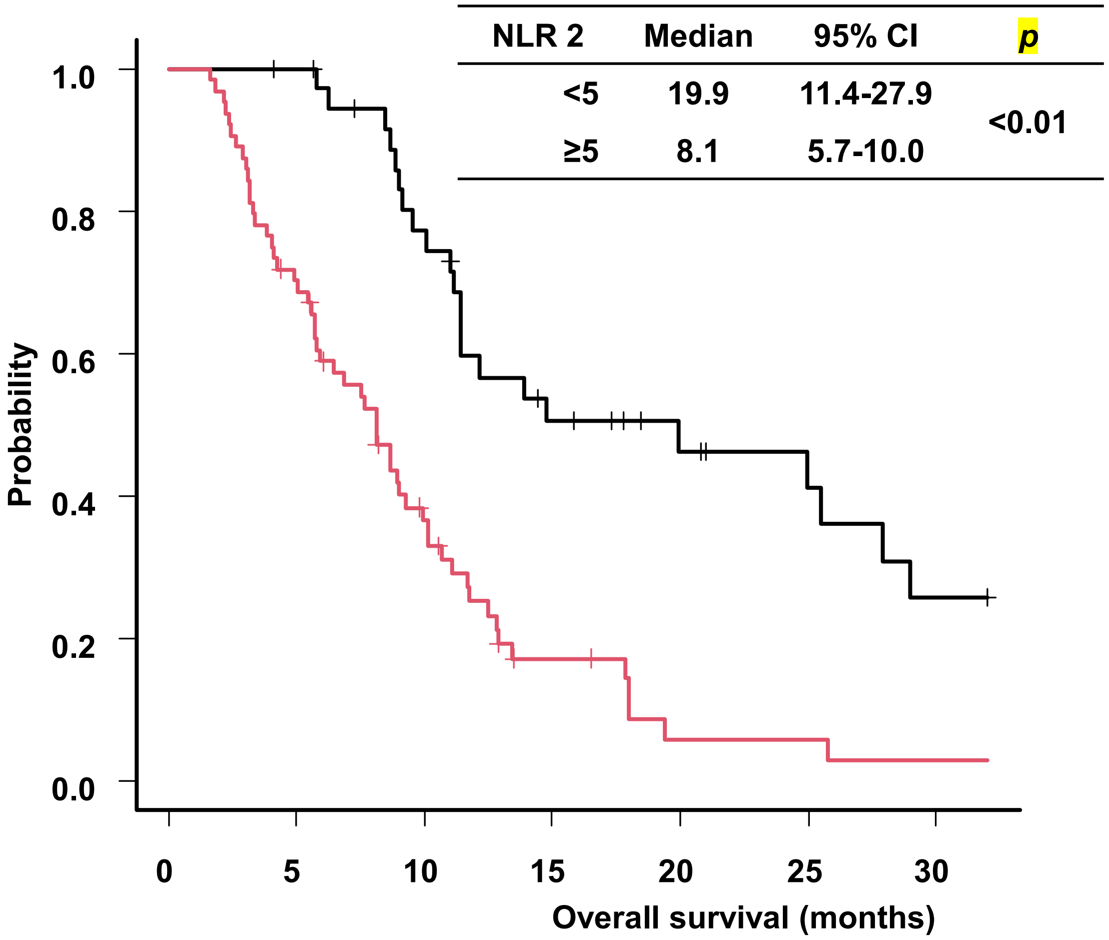

Number at risk

< 5	38	37	27	16	11	8	5
≥ 5	64	44	20	7	2	2	1

Figure S2.

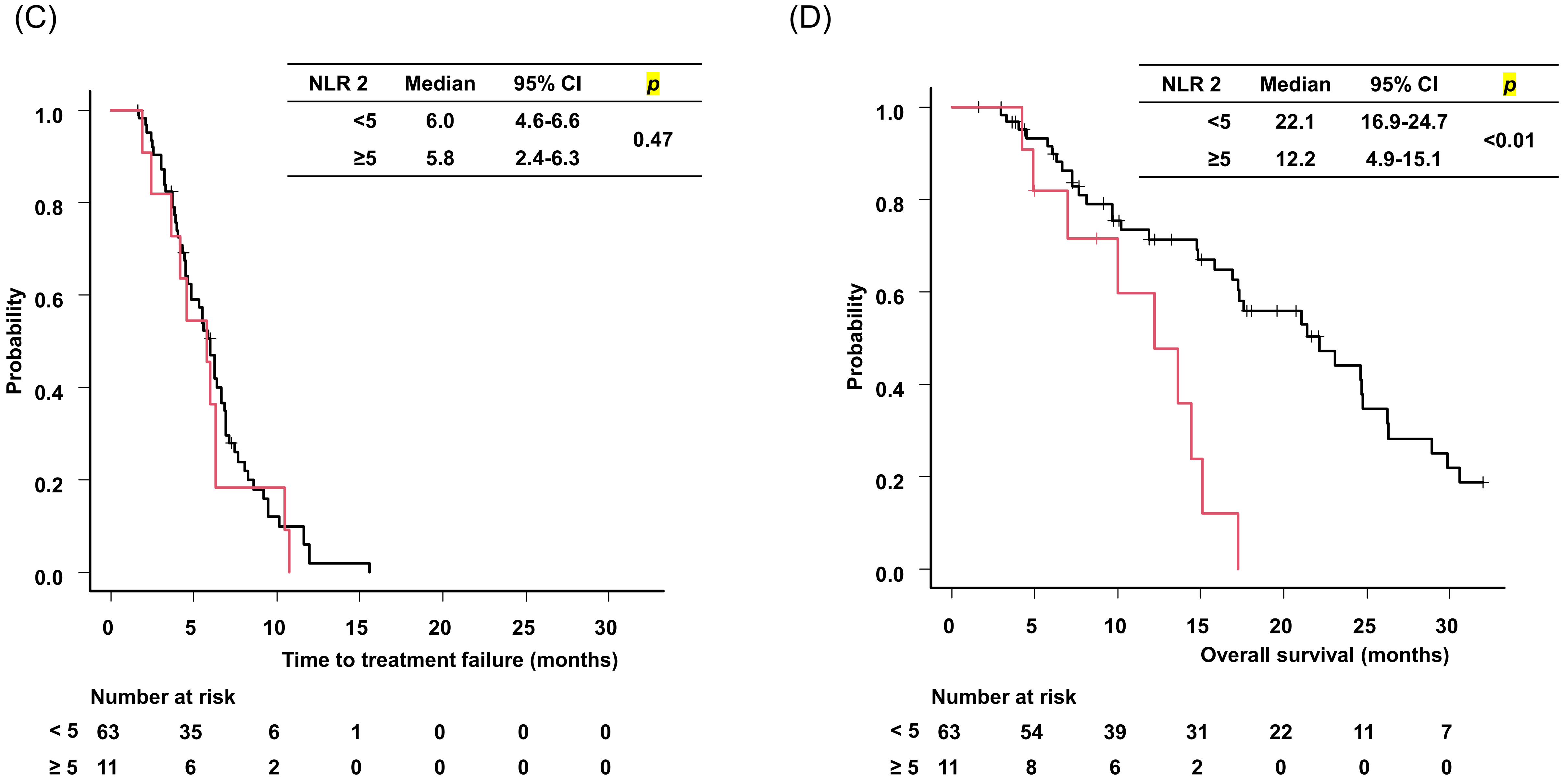
(C)
NLR 2
Median
95% CI
p
<5
6.0
4.6-6.6
≥5
5.8
2.4-6.3
0.47
Probability
1.0
0.8
0.6
0.4
0.2
0.0
0
5
10
15
20
25
30
Time to treatment failure (months)
Number at risk
< 5 63 35 6 1 0 0 0
≥ 5 11 6 2 0 0 0 0
(D)
NLR 2
Median
95% CI
p
<5
22.1
16.9-24.7
≥5
12.2
4.9-15.1
<0.01
Probability
1.0
0.8
0.6
0.4
0.2
0.0
0
5
10
15
20
25
30
Overall survival (months)
Number at risk
< 5 63 54 39 31 22 11 7
≥ 5 11 8 6 2 0 0 0

Figure S3.

(A)

Group	Median	95% CI	*p*
NLR <5, ΔNLR <0	6.3	4.5-7.6	<0.01
NLR <5 ΔNLR ≥0	4.9	4.2-6.0	
NLR ≥5, ΔNLR <0	4.8	4.2-6.3	
NLR ≥5, ΔNLR ≥0	2.3	1.9-3.1	

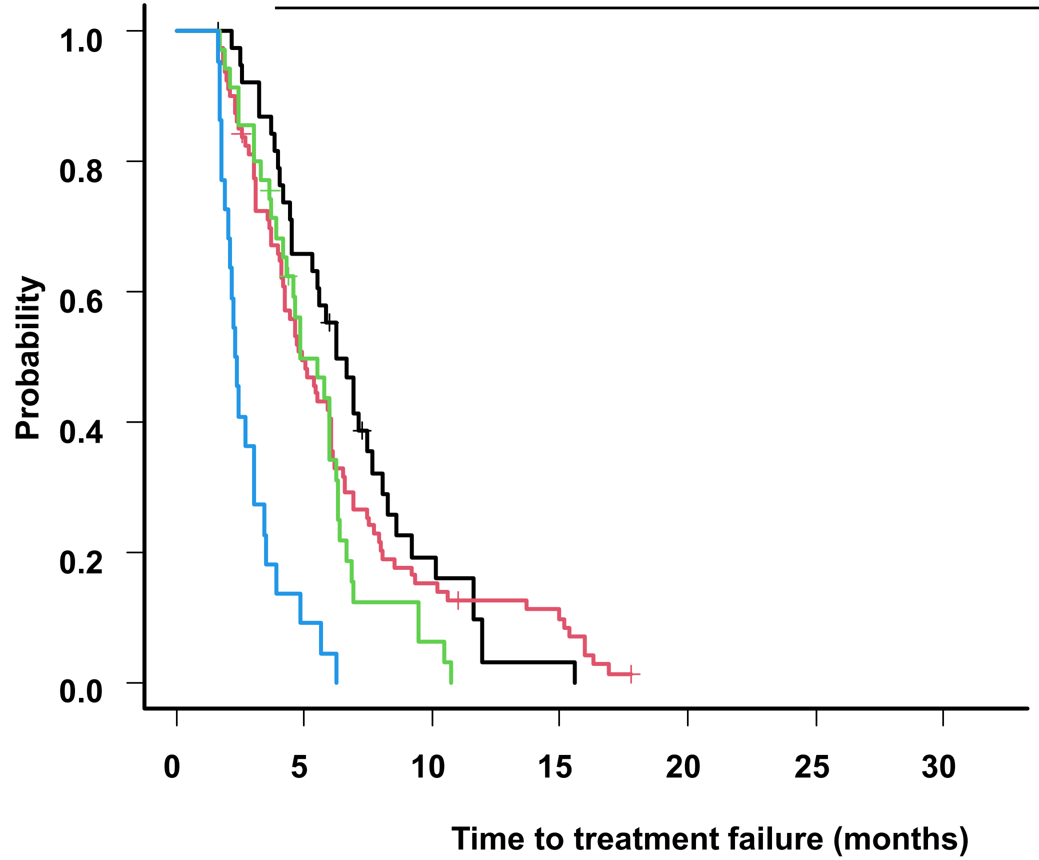

Number at risk

NLR <5, ΔNLR<0	39	25	6	1	0	0	0
NLR <5, ΔNLR≥0	80	39	12	7	0	0	0
NLR ≥5, ΔNLR<0	35	16	2	0	0	0	0
NLR ≥5 , ΔNLR≥0	22	2	0	0	0	0	0

(B)

Group	Median	95% CI	*p*
NLR <5, ΔNLR <0	22.2	16.9-26.2	<0.01
NLR <5 ΔNLR ≥0	11.4	10.1-13.9	
NLR ≥5, ΔNLR <0	13.6	7.7-17.3	
NLR ≥5, ΔNLR ≥0	4.0	3.0-5.8	

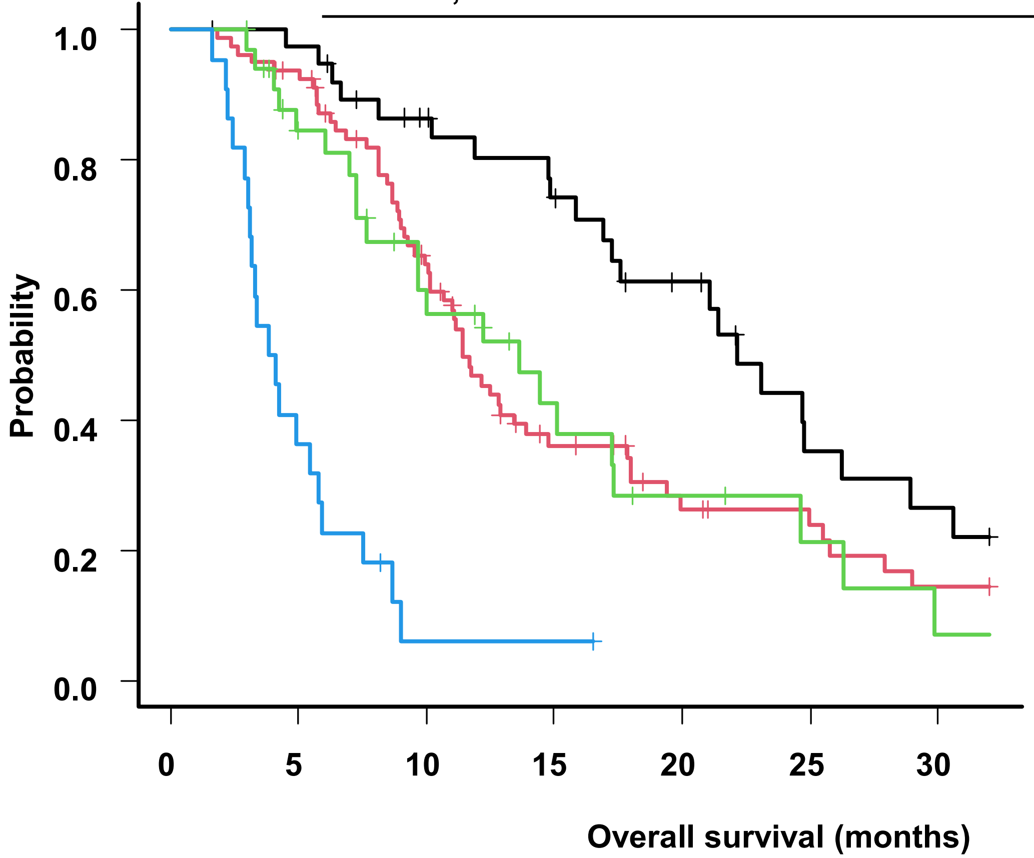

Number at risk

NLR <5, ΔNLR<0	39	37	29	24	17	8	6
NLR <5, ΔNLR≥0	80	73	46	22	13	10	6
NLR ≥5, ΔNLR<0	35	25	16	9	5	3	1
NLR ≥5 , ΔNLR≥0	22	8	1	1	0	0	0

Figure S4.

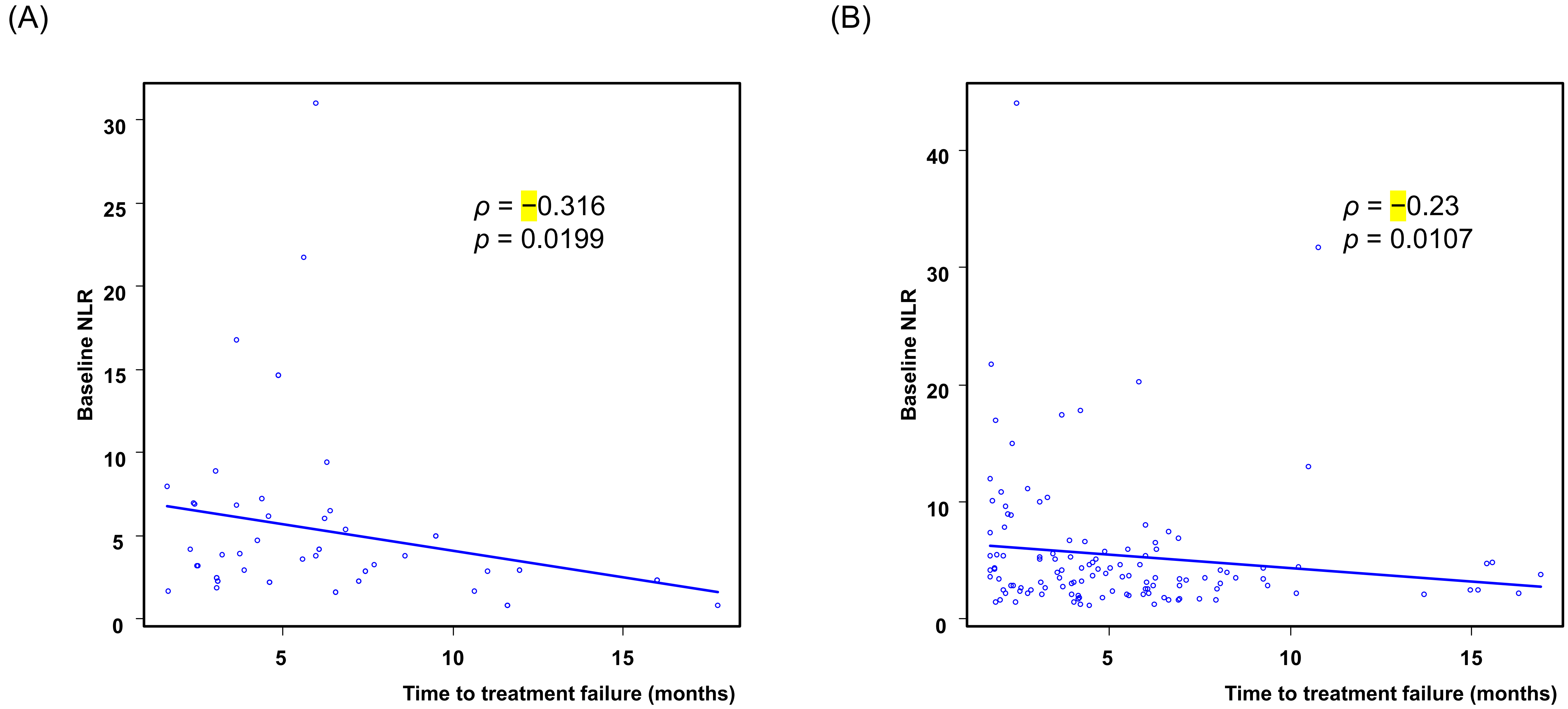
(A)
ρ = −0.316
p = 0.0199
Baseline NLR
Time to treatment failure (months)
0
5
10
15
20
25
30
5
10
15
(B)
ρ = −0.23
p = 0.0107
Baseline NLR
Time to treatment failure (months)
0
10
20
30
40
5
10
15

Figure S4.

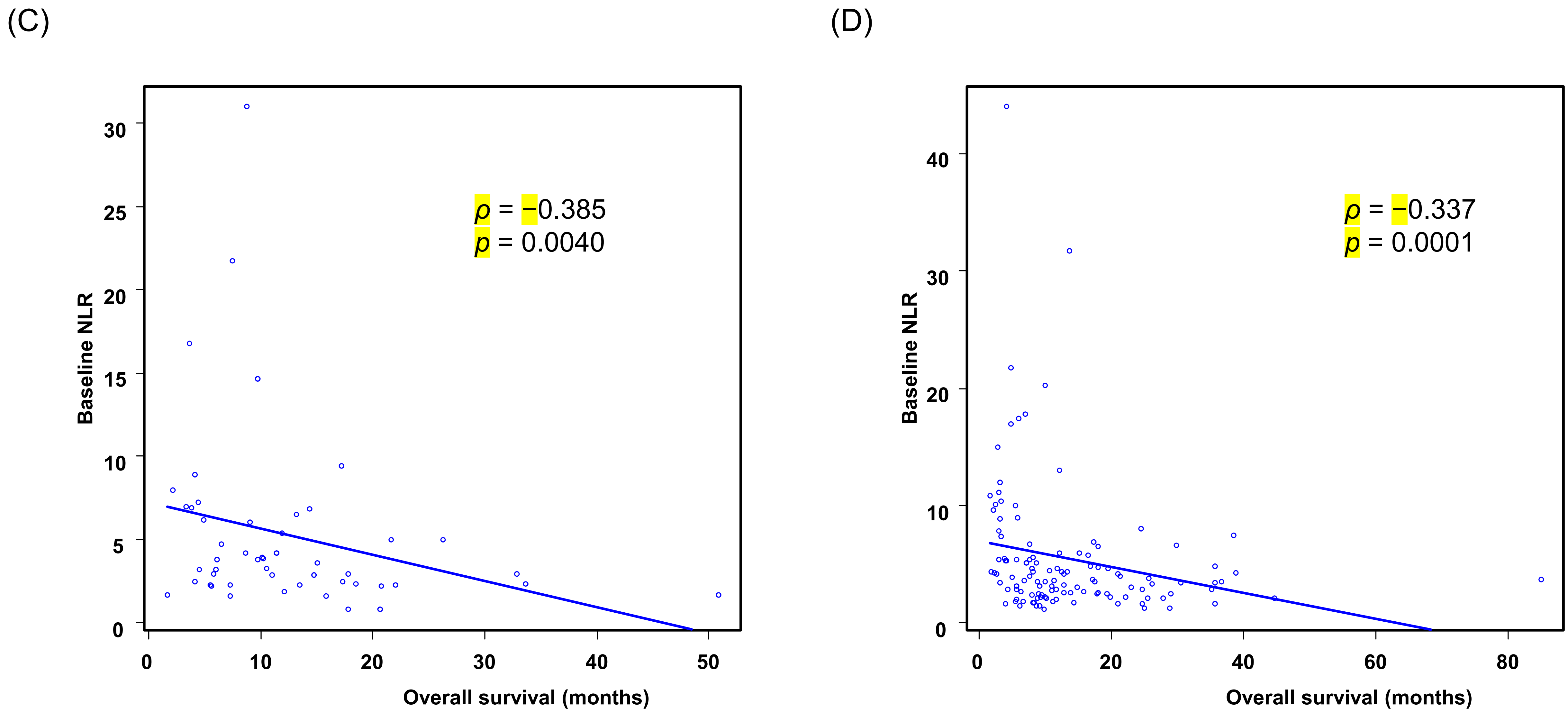
(C)
ρ = −0.385
p = 0.0040
Baseline NLR
0
5
10
15
20
25
30
0
10
20
30
40
50
Overall survival (months)
(D)
ρ = −0.337
p = 0.0001
Baseline NLR
0
10
20
30
40
0
20
40
60
80
Overall survival (months)
